# Human umbilical cord/placenta mesenchymal stem cell conditioned medium attenuates intestinal fibrosis in vivo and in vitro

**DOI:** 10.1186/s13287-024-03678-4

**Published:** 2024-03-07

**Authors:** Yoon Jeong Choi, Woo Ram Kim, Duk Hwan Kim, Jee Hyun Kim, Jun Hwan Yoo

**Affiliations:** 1 Department of Gastroenterology, CHA Bundang Medical Center, CHA University School of Medicine, 59 Yatap-ro, Bundang-gu, Seongnam, 13496 South Korea; 2 Institute of Basic Medical Sciences, CHA University School of Medicine, Seongnam, 13496 South Korea; 3 Department of Surgery, Gangnam Severance Hospital, Yonsei University College of Medicine, Seoul, 06273 South Korea

**Keywords:** Intestinal fibrosis, Conditioned medium, Mesenchymal stem cells, Umbilical cord, Placenta

## Abstract

**Background:**

A significant unmet need in inflammatory bowel disease is the lack of anti-fibrotic agents targeting intestinal fibrosis. This study aimed to investigate the anti-fibrogenic properties and mechanisms of the conditioned medium (CM) from human umbilical cord/placenta-derived mesenchymal stem cells (UC/PL-MSC-CM) in a murine intestinal fibrosis model and human primary intestinal myofibroblasts (HIMFs).

**Methods:**

UC/PL-MSC-CM was concentrated 15-fold using a 3 kDa cut-off filter. C57BL/6 mice aged 7 weeks old were randomly assigned to one of four groups: (1) control, (2) dextran sulfate sodium (DSS), (3) DSS + CM (late-phase treatment), and (4) DSS + CM (early-phase treatment). Chronic DSS colitis and intestinal fibrosis was induced by three cycles of DSS administration. One DSS cycle consisted of 7 days of oral DSS administration (1.75%, 2%, and 2.5% DSS), followed by 14 days of drinking water. UC/PL-MSC-CM was intraperitoneally administered in the late phase (from day 50, 10 times) or early phase (from day 29, 10 times) of DSS cycles. HIMFs were treated with TGF-β1 and co-treated with UC/PL-MSC-CM (10% of culture media) in the cellular model.

**Results:**

In the animal study, UC/PL-MSC-CM reduced submucosa/muscularis propria thickness and collagen deposition, which improved intestinal fibrosis in chronic DSS colitis. The UC/PL-MSC-CM significantly reduced the expressions of procollagen1A1 and α-smooth muscle actin, which DSS significantly elevated. The anti-fibrogenic effect was more apparent in the UC-MSC-CM or early-phase treatment model. The UC/PL-MSC-CM reduced procollagen1A1, fibronectin, and α-smooth muscle actin expression in HIMFs in the cellular model. The UC/PL-MSC-CM downregulated fibrogenesis by suppressing RhoA, MRTF-A, and SRF expression.

**Conclusions:**

Human UC/PL-MSC-CM inhibits TGF-β1-induced fibrogenic activation in HIMFs by blocking the Rho/MRTF/SRF pathway and chronic DSS colitis-induced intestinal fibrosis. Thus, it may be regarded as a novel candidate for stem cell-based therapy of intestinal fibrosis.

**Supplementary Information:**

The online version contains supplementary material available at 10.1186/s13287-024-03678-4.

## Background

Intestinal fibrosis is a significant complication of inflammatory bowel disease (IBD) and is more common in Crohn’s disease (CD) than in ulcerative colitis [1]. Over 50% of patients with CD exhibit fibrotic complications, most notably stricture and penetration, which result in potential intestinal obstruction and require surgical intervention [2]. Fibrostenosis caused by stricture formation remains a significant challenge in the management of patients with IBD, and few effective anti-fibrotic therapies are currently available. Most IBD treatments aim to reduce inflammation; however, treatments specifically targeting intestinal fibrosis are limited. Moreover, while it was long believed that suppressing chronic inflammation might prevent fibrosis, recent studies have revealed that it could persist even without inflammation [3]. Therefore, it is critical to develop anti-fibrotic agents that directly target intestinal fibrosis for the treatment of patients with CD and related gastrointestinal (GI) disorders.

The pathogenesis of intestinal fibrosis is a complex process that is not fully understood. Fibrosis is characterized by excessive deposition of extracellular matrix (ECM) proteins, including collagen and fibronectin, produced by activated myofibroblasts [4]. Activated myofibroblasts play a critical role in fibrosis development. These myofibroblasts express elevated levels of α-smooth muscle actin (α-SMA) and contribute to tissue distortion and intestinal strictures via ECM contraction [5]. Transforming growth factor (TGF-β1) is a crucial pro-fibrotic cytokine that stimulates fibrogenic myofibroblast activation [6, 7]. TGF-β1-induced fibrogenic activation occurs via Smad-dependent and -independent signaling pathways [8–10]. The RhoA/ROCK signaling pathway is critical in regulating actin cytoskeleton dynamics and transcriptional responses in numerous cell types, including fibrotic myofibroblasts. Activated RhoA triggers ROCK activation, promoting globular-actin (G-actin) polymerization into filamentous actin (F-actin), forming actin stress fibers and leading to contractile force generation by myofibroblasts. Moreover, the RhoA/ROCK signaling pathway regulates pro-fibrotic gene expression by promoting nuclear translocation of myocardin-related transcription factor A (MRTF-A), a co-activator of serum response factor (SRF), which is a transcription factor that regulates the expression of genes involved in cell proliferation and differentiation, including those that promote fibrosis [9, 11]. Further, when G-actin polymerizes into F-actin, MRTF-A is liberated from G-actin, allowing it to translocate to the nucleus and bind to SRF, thereby activating the transcription of pro-fibrotic genes.

Mesenchymal stem cells (MSCs) suppress the inflammatory response by secreting anti-inflammatory cytokines and regulating the immune system. They also have anti-fibrotic properties because they reduce the deposition of ECM and fibrosis-associated factors in tissues [12–16]. Several preclinical studies have found that MSCs can alleviate fibrosis in multiple organs, including the liver, lungs, kidneys, heart, skin, peritoneum, pancreas, and colorectum [17–22]. Umbilical cord and placenta-derived mesenchymal stem cells (UC/PL-MSCs) have gained attention as a promising alternative to traditional MSC populations for clinical applications owing to their ability to be obtained in larger quantities without invasive procedures and their higher proliferation and differentiation potential. Our previous study has revealed that UC/PL-MSCs have therapeutic potential by suppressing TGF-β1-induced fibrogenic activation in human primary intestinal myofibroblasts (HIMFs) [23].

The risk of vascular occlusion and malignant transformation following direct administration is important when considering the therapeutic use of MSCs [24–26]. There is increasing interest in investigating conditioned medium (CM) containing various biologically active molecules and extracellular vesicles (EVs) secreted by MSCs. Several enzymes involved in ECM-remodeling, such as matrix metalloproteinases (MMP2 and MMP9) and their inhibitors TIMP-1 and TIMP-2 have been found in MSC-CM [27–29]. In addition, anti-fibrogenic cytokines, such as interleukin-10 (IL-10) and prostaglandin-E2, as well as growth factors, such as vascular endothelial growth factor and hepatocyte growth factor (HGF), have been found. In addition to proteins, MSC-CM contains microRNAs with anti-fibrotic properties [30–32]. Owing to the ability of the CM to induce tissue regeneration and modulate the immune response, it is now considered a viable strategy for MSC-based therapy.

In this study, the anti-fibrotic effects and mechanisms of action of the CM derived from human UC/PL-MSCs (UC/PL-MSC-CM) in intestinal fibrosis were evaluated in a murine model of chronic colitis caused by dextran sodium sulfate (DSS) and in HIMFs. The results of this study provide new insights into the potential clinical use of UC/PL-MSC-derived products for treating fibrotic diseases of the intestine.

## Materials and methods

### Mice and reagents

Female C57BL/6 wild-type mice were purchased from Orient (Seongnam, Korea) and used under specific pathogen-free conditions. The mice were given assess to standard chow and sterile water ad libitum until they grew to the desired age (7–8 weeks) and body weight (19–22 g). DSS was purchased from MP Biochemicals (Irvine, CA, USA). Recombinant human TGF-β1 was obtained from R&D systems (Minneapolis, MN). The ethics committee at CHA University assessed and approved the procedures for conducting this animal study (IACUC210145). All experimental procedures in the animal study were performed in accordance with NIH guidelines.

### Preparation of human UC/PL-MSCs

CHA Biotech, Co. Ltd. (Seongnam, Korea) provided human umbilical cord- and placenta-derived MSCs. The isolation and expansion of human UC/PL-MSCs were performed according to the Good Clinical Practice guidelines of the Master Cell Bank. Further the preparations of the human UC/PL-MSCs were conducted in the Good manufacturing practices facility. The preparation and characterization of the cells have been described previously [23, 33–35]. Umbilical cord and placenta tissue were obtained with informed consent from healthy mother donors at CHA Bundang Medical Center (Seongnam, Korea). After severing the umbilical vessels, Wharton’s jelly was sliced into 1–5 mm explants to isolate UC-MSC. Isolated slices were attached to culture plates and subsequently cultured in α-modified minimal essential medium (α-MEM; Hyclone) supplemented with 10% fetal bovine serum (FBS; Gibco), 25 ng/mL fibroblast growth factor-4 (FGF4; Peprotech, London, England), 1 μg/mL heparin (Sigma, St. Louis, MO), and 0.5% gentamycin (Gibco) at 37 °C in a humidified atmosphere containing 5% CO_2_. Every 3 d, the medium was changed and on day 6, the UC-MSC cell populations emerged as outgrowths from the UC fragments. The umbilical cord fragments were discarded after 15 d, and the cells were grown until sub-confluence (80–90%) using TrypLE (Invitrogen, Carlsbad, CA). UC-MSCs at passage six were used, as described in our previous study, and the phenotype of the cells was determined using fluorescence-activated cell sorting (FACS) analysis [23].

The placental membranes were bluntly dissected from the placental body and then washed in Dulbecco’s phosphate-buffered saline (Gibco) to remove the blood from isolated the PL-MSCs. The amniotic connective tissue of the placental membranes was carefully harvested using two slide glasses and then incubated at 37 °C with shaking (175 rpm) for 15 min with HBSS containing 1 mg/mL type I collagenase (Sigma), 1.2 U/mL dispase (Gibco), 2 mg/mL trypsin (Sigma), 65 μg/mL DNase I (Roche, Mannheim, Germany), and 1 × penicillin–streptomycin (Gibco). The viability of the isolated cells was determined by trypan blue exclusion. PL-MSCs were cultured in α-MEM (Hyclone) supplemented with 10% FBS (Gibco), 25 ng/mL FGF4 (Peprotech), 1 μg/mL heparin (Sigma), and 0.5% gentamycin (Gibco) at 37 °C in a humidified atmosphere containing 5% CO_2_. FACS analysis was used to identify the phenotype of the cells, and the PL-MSCs at passage six were used in this study [23].

### Preparation of CM from human UC/PL-MSCs

A total of 1.0 × 10^6^ UC/PL-MSCs were seeded in a 100 mm dish (in 10 mL UC/PL-MSCs culture medium) to prepare the CM. The serum-free HIMFs culture medium was then replaced after 24 h. After incubating UC/PL-MSCs for 72 h, the CM was harvested. Cell debris was then removed by centrifuging the medium at 3800 × g for 10 min. The supernatant was collected, filtered through a 0.2 μm filter, and then concentrated 15-fold using a 3-kDa cut-off centrifugal filter (Amicon Ultra, Merck Millipore, Burlington, MA, USA) at 4000 × g for 40 min at 4 °C.

### Co-culture of HIMFs with UC/PL-MSC-CM

HIMFs grown in the presence of serum were starved for 24 h in HIMF serum-free medium before being treated with 5 ng/mL TGF-β1 alone or in combination with 10% UC/PL-MSC-CM to investigate the effects of UC/PL-MSC-CM on TGF-β1-mediated HIMFs fibrosis.

### Induction of DSS-induced fibrosis and UC/PL-MSC-CM treatment

**Fig. 1:**
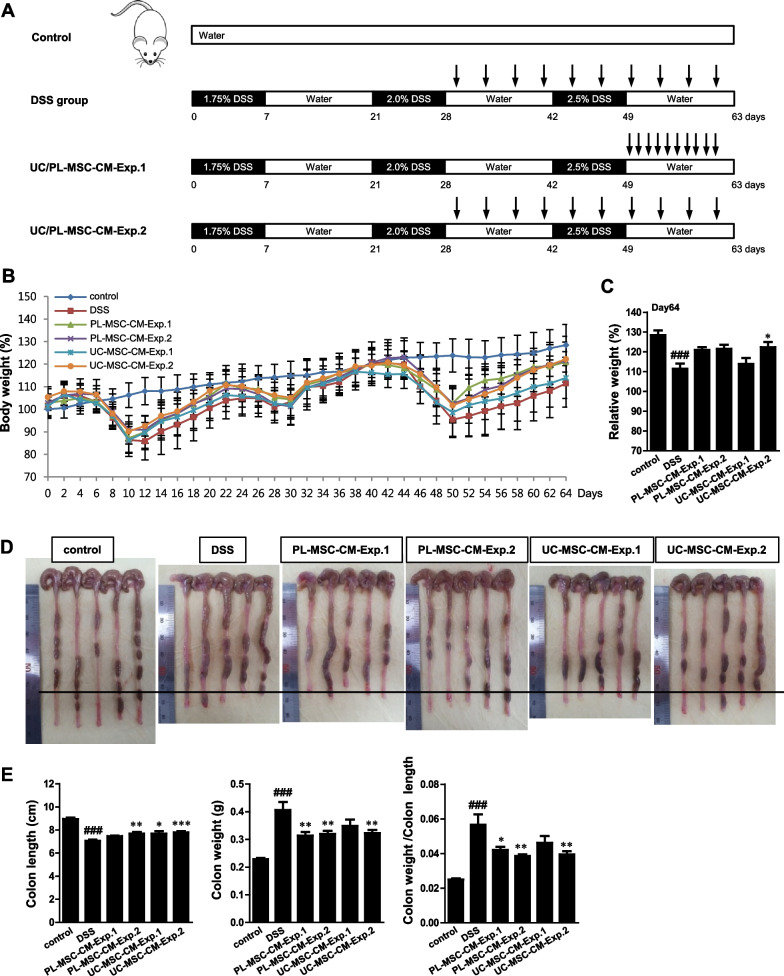
UC/PL-MSC-CM exhibits protective effects on the clinical course of chronic DSS-induced colitis.** A** Experimental design of repeated "cycles" of DSS exposure, as described in the "Methods" section. Mice were randomized into four groups: (1) control group (water-treated mouse group, n = 15); (2) DSS group (DSS and vehicle-treated mouse group, n = 15); (3) UC/PL-MSC-CM-Exp. 1 group (DSS and UC/PL-MSC-CM-treated mouse group according to Exp. 1 schedule, n = 10); and (4) UC/PL-MSC-CM-Exp. 2. (DSS and UC/PL-MSC-CM-treated mouse group according to Exp. 2 schedule, n = 15). Three DSS administration cycles induced chronic DSS colitis and intestinal fibrosis. One DSS cycle comprised of 7 d oral administration of DSS (1.75%, 2%, and 2.5% DSS), followed by 14 d of water drinking. In Exp. 1, the UC/PL-MSC-CM was injected intraperitoneally ten times during the late phase of the DSS cycles, starting from day 50. In Exp. 2, the UC/PL-MSC-CM was administered ten times during the early phase of the DSS cycles, starting from day 29. **B** Relative weight curve. **C** Relative weight at day 64. **D** Macroscopic appearance of the colons from the mice. **E** Colon length, colon weight, and colon weight/colon length of mice. Data are expressed as means ± SEM (n = 10–15 in each group). ^*###*^*P* < .001 versus the control group; ^*^P < .05, ^**^P < .01 and ^***^P < .001 versus the DSS group (ANOVA w/ Tukey)

Mice were subjected to repeated "cycles" of DSS administration to evaluate chronic colitis and fibrosis (Fig. 1A). One cycle was defined as a 7 d exposure to DSS, followed by a two-week recovery phase with normal drinking water. Mice were subjected to three cycles: one (1.75% DSS), two (2% DSS), and three (2.5% DSS). Control mice received normal drinking water. Two experimental groups were used to evaluate the anti-fibrotic effects of UC/PL-MSC-CM in an early- (Exp. 1) and late-phase treatment model (Exp. 2). In Exp. 1, we administered 150 μg/100 μL/mouse of UC/PL-MSC-CM intraperitoneally daily from Day 50 to Day 59. In Exp. 2, we administered 150 μg/100 μL/mouse of UC/PL-MSC-CM intraperitoneally twice weekly from Day 29 to Day 61. Four groups of mice were randomly assigned: (1) control group (water-treated mouse group, n = 15); (2) DSS group (DSS and vehicle-treated mouse group, n = 15); (3) UC/PL-MSC-CM-Exp. 1 group (DSS and UC/PL-MSC-CM-treated mouse group according to Exp. 1 schedule, n = 10); and (4) UC/PL-MSC-CM-Exp. 2 (DSS and UC/PL-MSC-CM-treated mouse group according to Exp. 2 schedule, n = 15). All experiments were performed in the same timeframe. On day 64, all mice were euthanized by CO_2_ asphyxiation.

### Macroscopic assessment and evaluation of fibrosis

Mice were weighed for body weight changes. After the mice were euthanized, the colon was removed entirely, and the length and weight were measured. The length from cecum to the anus was used to determine the length of the colon. The complete mouse colon was excised for historical analysis, and segments of the transverse tissues (1 cm) were then fixed in 10% buffered formalin, paraffin-embedded, and stained with hematoxylin and eosin. Zeiss Axio Scan.Z1 slide scanner was used to collect and digitize all histological sections, and ZEN 3.1 (blue edition) software was used for analysis. The thickness of the mucosa and muscularis propria of each mouse was calculated as the mean value of five distinct points. Sirius red staining (ab150681, abcam) was used to evaluate fibrosis. A hydroxyproline assay kit (ab222941, abcam) was used to obtain the collagen in the tissue according to the protocol of the manufacturer. A Zeiss Axio Scan.Z1 slide scanner was used to read the stained sections. Using ZEN 3.1 (blue edition) software, the quantified outcomes of Sirius red staining were presented as the mean density of five randomly selected fields from each group.

### Immunohistochemical analysis

Paraffin sections were deparaffinized in xylene and then hydrated in a graded ethanol series to evaluate the expression of Procol1A1 and α-SMA in colon tissues. REAL Peroxidase-Blocking Solution (S2023, DAKO, Carpinteria, CA, USA) was used to block the endogenous peroxidase activity for 30 min before masked antigens were retrieved using a 10 mM sodium citrate buffer at 95 °C for 1 h. Tissue slices were blocked with Protein Block solution (X0909, DAKO, Carpinteria, CA, USA) for 30 min before being incubated with a primary antibody at 4 °C overnight. The secondary antibody that was peroxidase-conjugated (#31430, Invitrogen) was incubated for 30 min at room temperature. All specimens were color developed using the DAB Substrate Kit (ab64238, Abcam, Cambridge, MA, USA) and counterstained with hematoxylin after being rinsed with PBS. The stained sections were read under a Zeiss Axio Scan.Z1 slide scanner and they were quantified using ZEN 3.1 (blue edition) software. The quantified results of Procol1A1 and α-SMA expression were presented as the mean density of five randomly selected fields from each group.

### Human intestinal myofibroblast isolation and culture

Primary HIMFs were isolated and cultured as per a previously described protocol with some modifications [36]. Briefly, HIMFs were derived from the outgrowths of minced colonic mucosa explants placed on etched polystyrene flasks containing HIMFs growth medium consisting of Dulbecco’s modified Eagle’s medium/high glucose (Hyclone, Logan, UT), 10% FBS (American Type Culture Collection, Manassas, VA), 4 mmol/L L-glutamine (Gibco, Carlsbad, CA), 25 mmol/L HEPES, 100 U/mL penicillin, 100 μg/mL streptomycin, and 0.25 μg/mL amphotericin B (all purchased from Lonza, Walkersville, MD) and used between passages 6 and 10 at 80% confluence. HIMFs were isolated from normal colon segments of patients undergoing resection for colorectal cancer. Following surgical resection, a pathologist removed the segments of the colon that were grossly normal from the area near the proximal resection margin. The periphery of the normal colon segments, which was used for isolation, was histologically confirmed under a microscope. The project was performed in accordance with the guidelines of the Institutional Review Board of the CHA Bundang Medical Center.

### RNA isolation and real-time quantitative PCR (RT-qPCR)

TRIzol reagent (Ambion, Carlsbad, CA) was used to extract RNA from the HIMFs. The ReverTra Ace qPCR RT Master Mix Kit (TOYOBO, Osaka, Japan) was then used to reverse-transcribe an identical amount of RNA (1 g) into cDNA according to the instructions of the manufacturer. All RT-qPCR reactions were performed using a Roche Light Cycler 96 instrument (Roche) with Faster-Start Essential DNA Probes Master (Roche). All gene mRNA levels were normalized to GAPDH levels. The specific primers for the collagen1A1 (Hs00164004_m1), fibronectin (Hs01549976_m1), α-SMA (ACTA2, Hs00426835_g1), Mkl1 (MRTF-A, Hs01090249_g1), SRF (Hs01065256_m1), RhoA (Hs00357608_m1), Rock1 (Hs01127701_m1), Rock2 (Hs00178154_m1), and GAPDH (Hs03929097_g1) genes were purchased from Applied Biosystems (Foster City, CA).

### Western blot

RIPA buffer (Cell Signaling, Beverly, MA) was used to isolate protein extracts. Protein samples were mixed with an equal volume of 5 × SDS sample buffer to separate on 10% SDS-PAGE gels after boiling for 5 min. The proteins were then transferred to polyvinylidene difluoride membranes following electrophoresis. Next, the membranes were blocked for 1 h at room temperature with 5% nonfat dry milk in Tris-buffered saline with Tween-20 buffer (TBS-T). Membranes were incubated with specific antibodies overnight at 4 °C. After three TBS-T washes to remove the primary antibodies, the membranes were treated for 2 h with horseradish peroxidase-conjugated anti-rabbit or anti-mouse immunoglobulin (GeneTex, Irvine, CA). Following three washes with TBS-T, antigen–antibody complexes were identified using the SuperSignal West Pico Chemiluminescence System (Thermo Fisher Scientific, Rockford, IL). A luminous image analyzer (ChemiDocTM XRS + System, Bio-Rad, USA) was used to acquire the signals. ImageJ 1.50i software (Wayne Rasband, National Institute of Health, USA) was used to quantify the western blots. The following antibodies were used: procollagen1A1 (Procol1A1) antibody (SP1D8, Developmental Studies Hybridoma Bank, Iowa City, IA) and fibronectin (FN) antibody (ab2413, Abcam, Cambridge, MA). Others include α-smooth muscle actin (α-SMA) antibody (A2547, Sigma), phospho-Smad2 (Ser465/467) antibody (#3108, Cell Signaling), RhoA antibody (#sc-418, Santa Cruz Biotechnology, Dallas, TX), GAPDH antibody (#2118, Cell Signaling), Mkl1(MRTF-A) antibody (21166-1-AP, ProteinTech), SRF antibody (#5147, Cell Signaling), and HDAC1 antibody (#5356, Cell Signaling).

### Immunocytochemistry

As previously mentioned, immunofluorescence staining was performed [7]. To evaluate the anti-fibrogenic properties of the UC/PL-MSC-CM, HIMFs (1.0 × 10^4^ cells/well) were seeded onto the chamber slides (#30108, SPL life Sciences, Korea) and exposed to TGF-β1 (5 ng/mL) co-incubated with or without UC/PL-MSC-CM for 48 h before being fixed with 4% paraformaldehyde for 10 min. The cells were treated with primary antibodies at 4 °C overnight after permeabilization with 0.1% Triton X-100 in 1 × PBS and blocking with 5% bovine serum albumin. The western blot contains information on all antibodies employed in immunocytochemistry. After three PBS washes (10 min each), the cells were incubated for 2 h at room temperature with AlexaFluor488/594-conjugated goat anti-mouse/rabbit secondary antibody (Molecular Probes, Eugene, OR). After washing, the cells were mounted using Fluoroshield Mounting Medium with DAPI (ab104139, Abcam) and examined using a Zeiss LSM880 confocal laser scanning microscope with the appropriate fluorescent filter.

Images were imported into the ImageJ software to quantify the nuclear-to-cytoplasmic ratio. Individual cells were defined with the Cell Mask dye, and the optical density of the MRTF-A staining was assessed and adjusted for cell area. The DAPI stain was then used to delineate the nucleus and calculate the density of the MRTF-A staining within it. Subtracting the nuclear fraction from the total cell calculation yielded the cytoplasmic fraction. The nuclear-to-cytoplasmic ratio was calculated by dividing the nuclear signal by the cytoplasmic signal.

### Extraction of nuclear and cytoplasmic proteins

Nuclear and cytoplasmic fractions of the HIMFs were extracted using the Nuclear Extraction kit (Millipore) according to the protocol of the manufacturer. The protein concentration was then determined using the bicinchoninic acid protein assay, followed by western blotting.

### Statistical analysis

The mean ± standard error of the mean of at least three independent experiments is used to express the data. The Mann–Whitney U test was used to compare the two groups. Analysis of variance with Tukey’s post hoc test was performed for multiple comparisons. Statistical significance was defined as *P*-values < 0.05. GraphPad Prism 8.0 (GraphPad, San Diego, CA, USA) was used for all statistical analyses.

## Results

### UC/PL-MSC-CM demonstrates protective effects on the clinical course of chronic DSS-induced colitis

A murine fibrosis model was constructed using repeated cycles of DSS exposure to explore the therapeutic effect of the human UC/PL-MSC-CM in chronic colitis. Two experiments, UC/PL-MSC-CM-Exp. 1 and UC/PL-MSC-CM-Exp. 2, were designed to assess the effects of UC/PL-MSC-CM treatment. In UC/PL-MSC-CM-Exp. 1, mice received daily intraperitoneal injections of UC/PL-MSC-CM (150 µg/100 µL/mouse) from Day 50 to Day 59, while UC/PL-MSC-CM-Exp. 2 involved twice-weekly intraperitoneal injections (150 µg/100 µL/mouse) from Day 29 to Day 61 (Fig. 1A). The findings revealed that weight loss in mice repeatedly exposed to DSS began on Day 6 and gradually recovered (Fig. 1B). Both UC/PL-MSC-CM-Exp. 1 and Exp. 2 groups exhibited less weight loss compared to the DSS group, with UC-MSC-CM-Exp. 2 showing a significant reduction in weight loss on Day 64 (Fig. 1C). In both Exp. 1 and Exp. 2 groups, treatment with PL-MSC-CM demonstrated a tendency towards a reduction in weight loss; however, this trend did not reach statistical significance. To evaluate the presence and extent of inflammation and fibrosis, the parameters of colon weight, length, and the weight-to-length ratio were systematically examined. DSS exposure significantly decreased colon length (Fig. 1D, E), indicating that DSS caused severe intestinal inflammation and fibrosis. However, in Exp. 2, UC/PL-MSC-CM treatment significantly reversed colon shortening in DSS-exposed mice. Furthermore, a significant increase in colon weight was observed in mice subjected to DSS treatment. In contrast, there was a notable decrease in colon weight in the PL-MSC-CM-Exp. 1 group and the UC/PL-MSC-CM-Exp. 2 group. Additionally, when compared with the DSS-treated group, the mice in both the PL-MSC-CM-Exp. 1 and UC/PL-MSC-CM-Exp. 2 groups exhibited significantly reduced colon weight-to-length ratios.

### UC/PL-MSC-CM ameliorates inflammation-associated intestinal fibrosis in chronic DSS-induced colitis

**Fig. 2:**
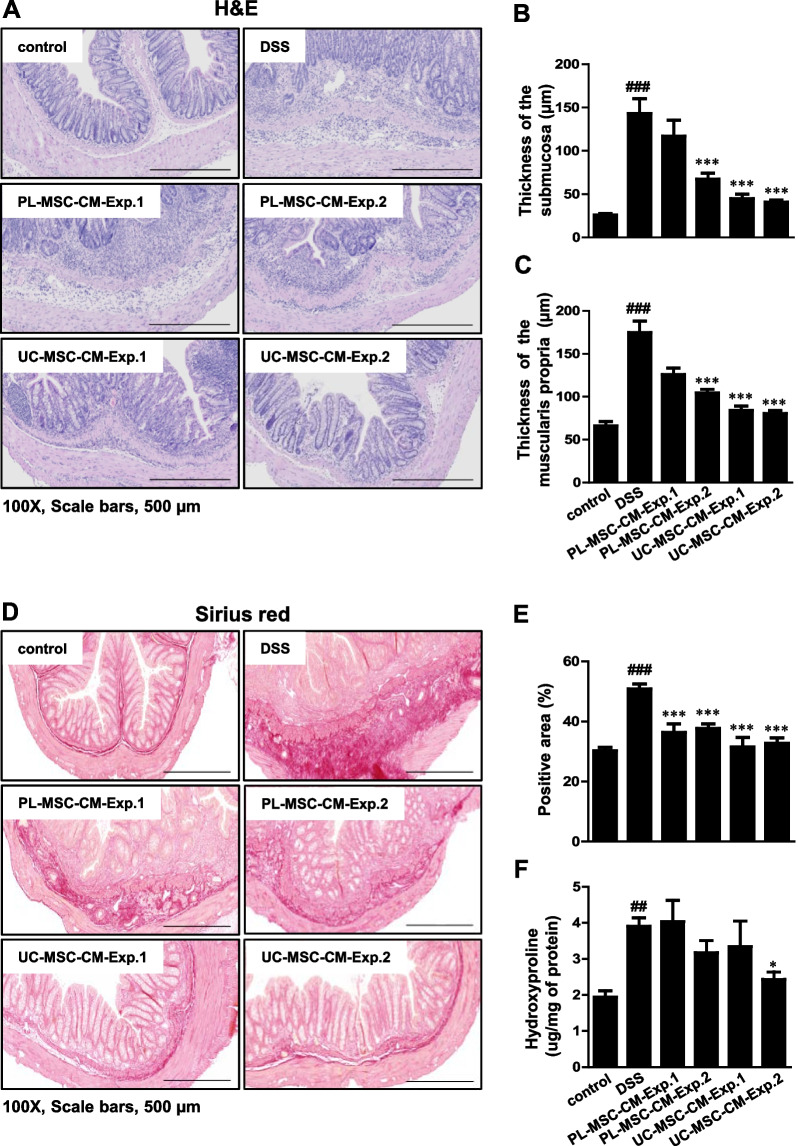
UC/PL-MSC-CM ameliorates inflammation-associated intestinal fibrosis in chronic DSS-induced colitis. **A** Representative hematoxylin and eosin [H&E] staining of colon sections. **B** Submucosal thickness (µm). **C** Muscularis propria thickness (µm). **D** Representative Sirius red staining of colon sections. **E** Positive area (%) in Sirius red staining. **F** Hydroxyproline concentration (µg/mg of protein). Data are expressed as means ± SEM (n = 10–15 in each group). ^##^*P* < .01 and ^###^*P* < .001 versus the control group; ^*^P < .05 and.^***^ *P* < .001 versus the DSS group (ANOVA w/ Tukey). Scale bars, 500 µm; original magnification, × 100 (**A, D**)

Chronic DSS exposure can cause chronic intestinal inflammation and lead to various complications, including aberrant epithelial structure, thicker gut wall, persistent collagen deposition, and mononuclear cell infiltration. Histological analysis showed that UC/PL-MSC-CM treatment significantly reduced colonic injury compared to the DSS group (Fig. 2A). Specifically, UC-MSC-CM-Exp. 1 and UC/PL-MSC-CM-Exp. 2 treatments protected the extension of crypt distortion while ameliorating inflammatory reactions such as mucosal and submucosal infiltrations. Furthermore, the thickness of the submucosa and muscularis propria increased significantly in the DSS group, followed by a significant decrease after treatment with UC-MSC-CM in Exp. 1 and UC/PL-MSC-CM in Exp. 2 (Fig. 2B, C).

Sirius red staining, a technique for histological analysis, was employed to visualize the accumulation of collagen in the mucosal and submucosal layers, indicative of fibrosis, in mice with chronic colitis. The results showed that administration of UC/PL-MSC-CM in Exp. 1 and Exp. 2 had a protective effect against fibrosis-associated collagen deposition (Fig. 2D, E). In addition, we assessed the levels of hydroxyproline (collagen metabolites) to assess the severity of colon fibrosis. The hydroxyproline concentration was found to be significantly higher in the DSS-treated group when compared to the control group. Conversely, in the UC-MSC-CM-Exp. 2 group, this concentration was significantly lower (Fig. 2F).

**Fig. 3:**
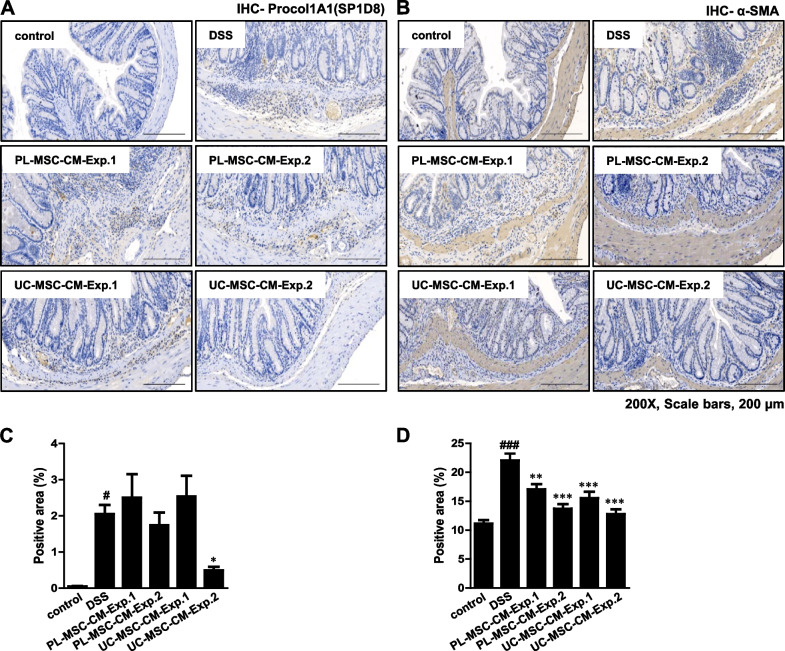
Immunohistochemical (IHC) staining of Procol1A1 and α-SMA. **A** Representative IHC staining of Procol1A1. **B** Representative IHC staining of α-SMA. **C** Procol1A1 quantification by IHC staining of the colon. **D** α-SMA quantification by IHC staining of the colon. Data are presented as mean ± SEM. ^#^*P* < .05 and ^###^*P* < .001 versus the control group. ^*^P < .05, ^**^P < .01, and.^***^P < .001 versus the DSS group (ANOVA w/ Tukey). Scale bars, 200 µm; original magnification, × 200 (**A, B**)

Biomarkers such as Procol1A1 and α-SMA are commonly utilized for the evaluation of intestinal fibrosis. The administration of DSS resulted in a significant increase in the expression levels of both Procol1A1 and α-SMA, as shown in Fig. 3. Notably, in Exp. 2, treatment with UC-MSC-CM led to a significant reduction in Procol1A1 expression (Fig. 3A, C). Furthermore, the expression of α-SMA was significantly decreased following UC/PL-MSC-CM treatment in both Exp. 1 and Exp. 2 (Fig. 3B, D). RT-qPCR analysis was used to evaluate the colonic mRNA expression of fibrosis-related genes (*Col1a1, Fn1, Acta2, Col3a1, Tnfa, Tgfb1, Ccn2, Mmp2, Mmp9,* and *Timp1*) (Additional file 1: Fig. S1 and Additional file 2). In Exp. 2, UC-MSC-CM significantly reduced the rise in *Tnfa* mRNA expression observed in the DSS group. In Exp. 2, treatment with UC-MSC-CM exhibited a trend towards the reduction in the mRNA expression levels of *Acta2, Tgfb1, Mmp9, and Timp1*. However, these observed changes did not achieve statistical significance. These observations lend further support to the hypothesis that UC/PL-MSC-CM has a beneficial effect on intestinal fibrosis associated with chronic colitis.

### UC/PL-MSC-CM inhibits TGF-β1-induced ECM and α-SMA expression in human intestinal myofibroblasts

**Fig. 4:**
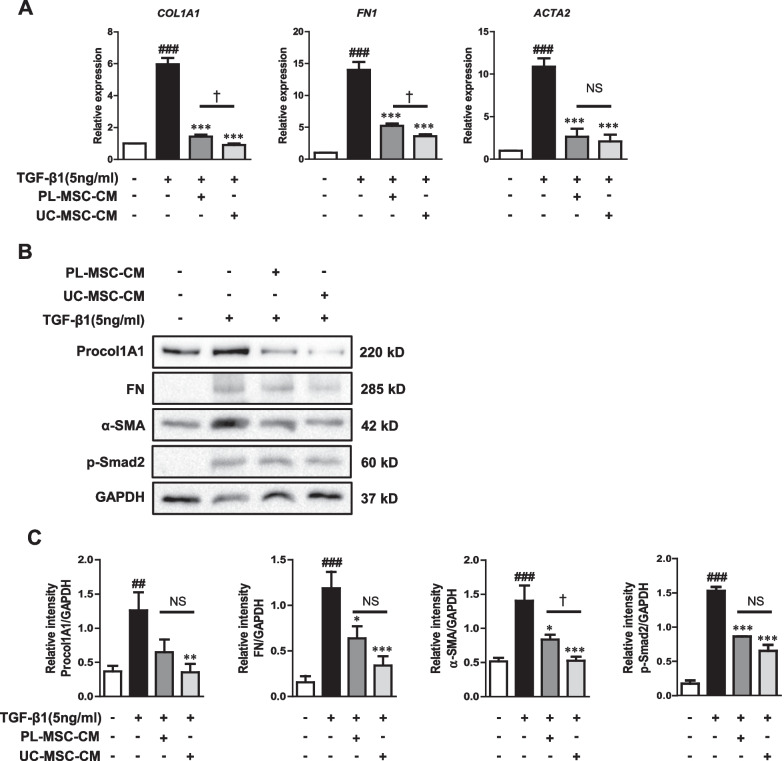
Co-culture with UC/PL-MSC-CM inhibits TGF-β1-induced fibrogenic activation of HIMFs. HIMFs were treated with TGF-β1 (5 ng/mL) and co-cultured with or without UC/PL-MSC-CM. **A** RT-qPCR analysis of the relative mRNA expression of collagen1A1 (*COL1A1*), fibronectin (*FN1*), and α-smooth muscle actin (*ACTA2*). The data were normalized to GAPDH expression and expressed as relative values compared to the control (n = 3). **B** Representative Western blots show the protein expression of procollagen1A1 (Procol1A1), fibronectin (FN), α-smooth muscle actin (α-SMA), and phosphorylated Smad2 (p-Smad2) with GAPDH as a loading control. **C** Quantitation of Procol1A1, FN, α-SMA, and p-Smad2 from Western blot analyses (n = 4). Data are expressed as means ± SEM. ^##^*P* < .01 and ^###^*P* < .001 versus the control; ^*^P < .05, ^**^P < .01, and ^***^P < .001 versus the TGF-β1 treatment only (ANOVA w/ Tukey); ^†^*P* < .05 compared between PL-MSC-CM and UC-MSC-CM (Mann–Whitney U test). NS, not significant. Full-length blots are presented in Additional file 1: Fig. S3

HIMFs were co-cultured with UC/PL-MSC-CM and simultaneously stimulated with TGF-β1 to evaluate the anti-fibrotic effects of human UC/PL-MSC-CM on myofibroblasts. TGF-β1 significantly increased mRNA expression of collagen1A1 (*COL1A1*), FN (*FN1*), and α-SMA (*ACTA2*) in HIMFs. In contrast, co-culture with UC/PL-MSC-CM significantly inhibited this effect in a dose-dependent manner (Fig. 4A, Additional file 1: Fig. S2 and Additional file 2). Notably, the comparison between UC-MSC-CM and PL-MSC-CM treatments revealed no significant difference in the expression levels of *ACTA2* mRNA. However, a more pronounced reduction in the expression of *COL1A1* and *FN1* mRNA was observed with the application of UC-MSC-CM. At the protein level, treatment with UC-MSC-CM resulted in a significant reduction of the TGF-β1-induced increase in Procol1A1 protein expression. In contrast, while PL-MSC-CM treatment also displayed a trend towards decreased Procol1A1 protein expression, this reduction did not reach statistical significance (Fig. 4B, C; Additional file 1: Fig. S3). UC/PL-MSC-CM also significantly reduced the TGF-β1-induced upregulation of FN and α-SMA protein expression. Contrary to the trends observed at the mRNA expression levels, the reduction in Procol1A1 and FN protein expression did not exhibit a statistically significant difference between the UC-MSC-CM and PL-MSC-CM treatments. However, the reduction in α-SMA expression was significantly more pronounced in the UC-MSC-CM treatment group. In addition, the UC/PL-MSC-CM treatment significantly attenuated the Smad2 phosphorylation induced by TGF-β1 (Fig. 4B, C). This suggests that the anti-fibrogenic effects of UC/PL-MSC-CM may be mediated through Smad-dependent pathways.

**Fig. 5:**
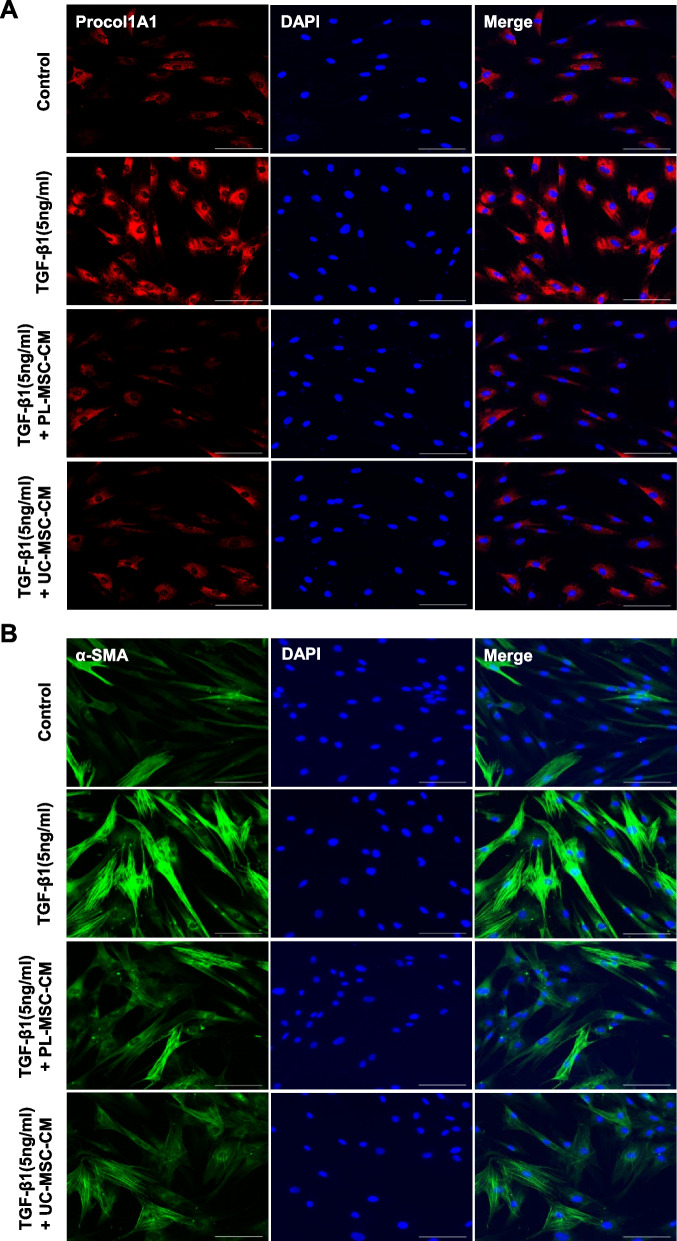
Co-culture with UC/PL-MSC-CM inhibits TGF-β1-induced Procol1A1 and α-SMA expression in HIMFs.** A–B** HIMFs were treated with TGF-β1 (5 ng/mL) and co-cultured with or without UC/PL-MSC-CM and then stained with Procol1A1 (**A)** and α-SMA (**B**) antibodies and counterstained with DAPI. Scale bars, 100 μm; original magnification, × 200

Immunostaining of Procol1A1 and α-SMA in HIMFs treated with TGF-β1 revealed a significant increase in Procol1A1 staining and the presence of well-organized, intensely stained actin stress fibers (Fig. 5A, B). However, treatment with UC/PL-MSC-CM resulted in a reduction of Procol1A1 staining and produced a diffuse, less intense α-SMA staining, similar to that observed in control samples. These findings suggest that both UC- and PL-MSC-CM are capable of effectively inhibiting the fibrogenic activation of HIMFs induced by TGF-β1, as evidenced by the downregulation of ECM components and α-SMA at both the mRNA and protein levels.

### UC/PL-MSC-CM inhibits the Rho/MRTF/SRF signaling in human intestinal myofibroblasts.

**Fig. 6:**
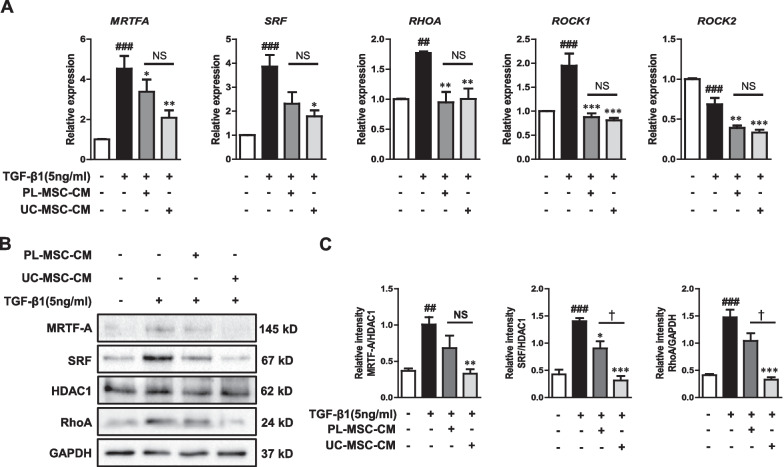
UC/PL-MSC-CM inhibits Rho/MRTF/SRF signaling in HIMFs. HIMFs were treated with TGF-β1 (5 ng/mL) and co-cultured with or without UC/PL-MSC-CM. **A** RT-qPCR analysis of the relative mRNA expression of *MRTFA, SRF, RHOA, ROCK1*, and *ROCK2*. The data were normalized to GAPDH expression and expressed as relative values compared to the control (n = 3). **B** Representative western blots show the protein expression of MRTF-A, SRF, and RhoA (MRTF-A and SRF from the nuclear extracts with HDAC1 as a loading control and RhoA from the cytosolic extracts with GAPDH as a loading control). **C** Quantitation of MRTF-A, SRF, and RhoA from western blot analyses (n = 3). Data are expressed as the means ± SEM. ^##^*P* < .01 and ^###^*P* < .001 versus the control; ^*^P < .05, ^**^P < .01 and ^***^P < .001 versus the TGF-β1 treatment only (ANOVA w/ Tukey); ^†^*P* < .05 compared between PL-MSC-CM and UC-MSC-CM (Mann–Whitney U test). NS, not significant. Full-length blots are presented in Additional file 1: Fig. S4

We investigated the role of the Rho/MRTF/SRF signaling pathway in the anti-fibrotic effects of UC/PL-MSC-CM. UC/PL-MSC-CM significantly reduced *MRTFA* mRNA expression induced by TGF-β1 in HIMFs, while only UC-MSC-CM significantly reduced *SRF* mRNA expression (Fig. 6A). We also examined the expression of upstream signaling molecules, including *RHOA*, *ROCK1*, and *ROCK2*, which have been found to be important in fibrosis development in most organs [37]. The mRNA expression of *RHOA* and *ROCK1* induced by TGF-β1 in HIMFs was significantly reduced by co-culture with UC/PL-MSC-CM, with no significant difference observed between the UC-MSC-CM and PL-MSC-CM. In addition, the expression level of *ROCK2* mRNA was significantly reduced in the co-culture treated with UC/PL-MSC-CM compared to the treatment with TGF-β1 alone. Contrary to *ROCK1*, there was a decrease in *ROCK2* mRNA expression in response to TGF-β1 treatment (Fig. 6A).

At the protein level, treatment with UC-MSC-CM resulted in a significant reduction in the expression of MRTF-A and SRF in nuclear extracts, as well as RhoA in cytosolic extracts. All of these proteins had previously been stimulated by TGF-β1 (Fig. 6B, C; Additional file 1: Fig. S4). Treatment with PL-MSC-CM led to a significant reduction in SRF expression, while also showing a downward trend in the expression levels of MRTF-A and RhoA. Furthermore, the extent of reduction in SRF and RhoA expressions was significantly more pronounced in the UC-MSC-CM treatment compared to the PL-MSC-CM (Fig. 6B, C; Additional file 1: Fig. S4).

**Fig. 7:**
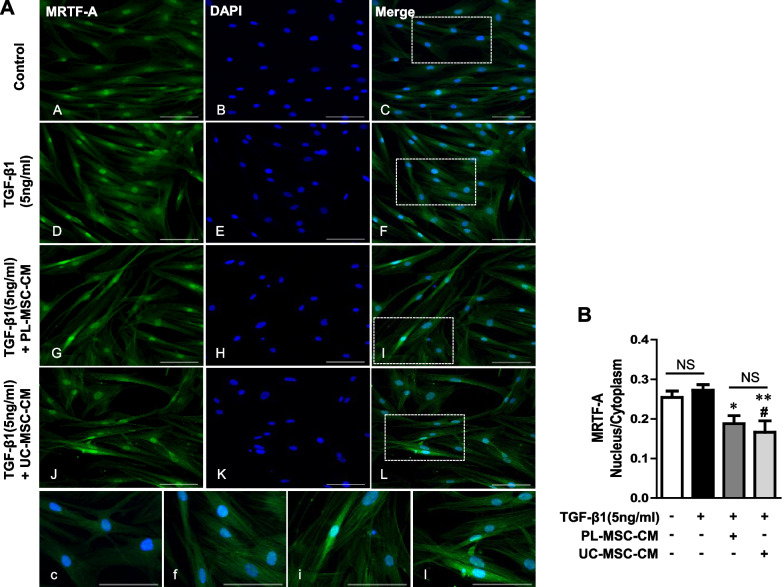
UC/PL-MSC-CM inhibits MRTF-A nuclear localization in HIMFs. HIMFs were treated with TGF-β1 (5 ng/mL), co-cultured with or without UC/PL-MSC-CM, stained with MRTF-A antibodies, and counterstained with DAPI. **A** (A–C) no treatment; (D–F) treatment with TGF-β1; (G–I) treatment with TGF-β1 and PL-MSC-CM; (J–L) treatment with TGF-β1 and UC-MSC-CM; (c, f, i, l) Enlarged images of the region (within the white box of C, F, I, and L, each). **B** The nuclear-to-cytoplasmic ratio of MRTF-A was determined for each condition as described in the "Methods" section. Data are expressed as means ± SEM. ^#^*P* < .05 versus the control; ^*^P < .05 and ^**^P < .01 versus the TGF-β1 treatment only (ANOVA w/ Tukey). NS, not significant (Mann–Whitney U test); Scale bars, 100 μm; original magnification, × 200 (**A**)

To further investigate the mechanisms underlying the anti-fibrotic properties of UC/PL-MSC-CM, we explored the potential role of MRTF-A nuclear localization inhibition in this anti-fibrotic process. This involved a specific quantification of the nuclear-to-cytoplasmic signal ratio in MRTF-A immunocytochemistry. The application of UC/PL-MSC-CM was associated with a significant decrease in MRTF-A nuclear localization (Fig. 7A, B). These results suggest that UC/PL-MSC-CM may exert its anti-fibrotic effects by inhibiting Rho/MRTF/SRF signaling pathways in HIMFs.

## Discussion

Our in vivo study revealed that UC/PL-MSC-CM alleviated inflammation-associated intestinal fibrosis in chronic DSS-induced colitis. Through the downregulation of Procol1A1 and α-SMA, UC/PL-MSC-CM treatment prevented the development of intestinal fibrosis in chronic colitis produced by repeated cycles of DSS exposure. The anti-fibrotic effect was more significant in the UC-MSC-CM group or in the early-phase treatment model (Exp. 2), where the CM was administered twice weekly starting from day 29. Furthermore, our in vitro results showed that UC/PL-MSC-CM inhibits TGF-β1-induced ECM (Procol1A1 and FN) synthesis and α-SMA formation in HIMFs by blocking Rho/MRTF/SRF signaling. These findings suggest that UC/PL-MSC-CM is a promising choice for stem cell-based therapy of intestinal fibrosis.

MSCs exhibit anti-fibrotic properties, suggesting that they may have therapeutic implications for fibrotic diseases [38–41]. Although research in this area is still limited, there is evidence that MSCs, including UC/PL-MSCs, are promising for the prevention and treatment of fibrogenesis, particularly in intestinal fibrosis [23, 42]. However, various issues must be addressed, such as MSC heterogeneity, short lifespan, and potential tumorigenic risks, especially when used in high doses or after genetic modification [43–45]. In terms of therapy, MSCs primarily operate through paracrine signaling, releasing a mixture of soluble factors and EVs into their CM. The CM, which combines these elements, offers advantages over whole MSCs, including reduced risks of tumorigenicity and immunological issues and ease of storage [46–48]. Furthermore, CM production is more cost-effective and efficient than EV isolation; CM benefits from high concentrations of soluble factors and EVs, particularly from adipose-derived MSCs [49]. Studies have shown that MSC CM and EVs contain anti-fibrogenic agents, like HGF, TGF-β3, IL-10, MFGE-8, miR-27b, and miR-223-3p, which are capable of blocking the TGF-β1/Smad2/3 signaling pathway and thereby inhibiting the transition of fibroblasts to myofibroblasts [31, 32, 50–55]. Hence, MSC-CM has emerged as a potentially useful therapeutic product.

Our in vivo study demonstrated that UC/PL-MSC-CM ameliorates intestinal fibrosis, as evidenced by reductions of collagen and α-SMA deposition based on measurements of the thickness of the submucosa and muscularis propria, Sirius red staining, hydroxyproline quantification, and Procol1A1 and α-SMA immunohistochemistry. Few studies have evaluated the MSC-CM in animal models of intestinal fibrosis. The effect of MSCs engineered to overexpress hypoxia-inducible factor 1-alpha and telomerase (MSC-T-HIF) conditioned with pro-inflammatory stimuli to release EVs (EVMSC-T-HIFC) on the fibrosis and inflammatory response of activated endothelium was examined in a recent study investigating experimental CD [56]. EVMSC-T-HIFC administration to mice with acute TNBS-induced colitis accelerated mucosal healing, reduced inflammation, and alleviated intestinal fibrosis. A recent study in 2023 has shed light on the therapeutic potential of human UC MSC-derived exosomes (hucMSC-Ex) in the context of IBD-associated intestinal fibrosis. In animal models, hucMSC-Ex reduced inflammation-related fibrosis, inhibited TGF-β-induced proliferation, migration, and activation of human intestinal fibroblasts, and decreased ERK phosphorylation, a key process in IBD-associated fibrosis [57]. Another study conducted in 2023 has revealed that the intratracheal application of MSC-EVs plays a pivotal role in the amelioration of established pulmonary fibrosis; this therapeutic effect can primarily be attributed to the transfer of specific microRNAs encapsulated within MSC-EVs [58]. Administration of the MSC culture supernatant significantly reduced the degree of luminal stricture in the rectum and mitigated myofibroblast activation and hypertrophy of the muscularis propria in pigs [59]. Various challenges remain in the field of MSC-based cell-free therapies for intestinal fibrosis, including an insufficient understanding of the MSC secretome and its intercellular interactions as well as the need to optimize the administration of MSC-derived components in terms of dosage, timing, and delivery methods.

MSC-EVs/exosomes exhibit the capacity to migrate to inflamed tissues, where they effectively interact with and modulate immune cells, actively regulating immune responses and exerting anti-inflammatory effects [60, 61]. In our study, we did not conduct live cell tracing to evaluate the migration of MSC-CM to the fibrotic area. Nevertheless, prior research provides some insights that might be pertinent to our findings. For example, a study published in 2015 demonstrated that intraperitoneally injected exosomes migrate to several organs, including the liver, lung, spleen, pancreas, and GI tract [62]. Of particular note in the context of the GI tract, exosome accumulation was significantly higher after intraperitoneal injection than after intravenous injection. Furthermore, the quantity of exosomes in the GI tract was comparable to that in the liver. These findings suggest that the intraperitoneal administration of exosomes is a promising therapeutic strategy for GI tract diseases [63]. A plausible interpretation of these findings is that lymphatic vessels lack a basement membrane, resulting in considerably greater permeability, enabling the diffusion of large molecules. Building upon this premise, peritoneally infused exosomes could be readily absorbed by mesenteric or peritoneal lymphatics. Following uptake, these exosomes have the potential to migrate to various organs, including the GI tract [64].

Furthermore, intraperitoneally injected MSC-CM may induce anti-inflammatory and anti-fibrotic effects within the mesenteric fat. Accumulating evidence suggests that there is a connection between changes in the mesenteric fat and CD [65]. Intestinal barrier dysfunction and transmural inflammation may induce bacterial translocation to the surrounding mesenteric adipose tissue, which subsequently leads to adipocyte hypertrophy. The hypertrophic adipocytes release pro-inflammatory mediators, which can activate the infiltration of M1 macrophages and Th1 and Th17 cells into mesenteric fat [66, 67]. This process may contribute to the development of hypertrophic mesenteric fat wrapping around the inflamed intestine, known as “creeping fat,” which is pathognomonic of CD [65]. The creeping fat in CD is associated with intestinal fibrosis by releasing broad spectrum of profibrotic mediators [68, 69]. MSC-CM contains a range of soluble anti-inflammatory factors, such as IL-10 and TGF-β. Upon intraperitoneal injection, these soluble factors can reach the mesenteric fat and may inhibit the activity of pro-fibrotic cells, such as M1 macrophages and Th1 and Th17 cells, within the mesenteric fat. The observed antifibrotic potential of intraperitoneally injected MSC-CM could be attributed to its influence on both innate and adaptive immune cells. This hypothesis is supported by a study of a DSS-induced colitis model, where UC-MSC-CM facilitated the polarization of M1 macrophages into an M2 phenotype. This shift was associated with a decrease in pro-fibrotic cytokines (IL-1β, MCP-1, TIMP-1, and IL-17) and an increase in the anti-fibrotic IL-10 in the colon or peritoneum [70]. Complementing these findings, Heidari et al. reported that UC-MSC exosomes prompt a similar immunomodulatory effect in a mouse model of DSS-induced colitis. They observed a Th1/Th17 polarization of regulatory T cells, leading to the reduced expression of pro-fibrotic mediators (TNFα, IL-17) and enhanced expression of the anti-fibrotic mediator IL-10 in mesenteric lymph nodes [71].

Additional file 1: Fig. S2 summarizes the mRNA expression levels of other fibrosis-related genes. In Exp. 2, the proinflammatory and profibrotic cytokine *Tnfa* was significantly reduced in the colon tissues of UC-MSC-CM-treated mice, consistent with the findings of previous studies, indicating that MSC-CM/EVs suppress TNF-α expression in a DSS colitis model [72–74]. Furthermore, in Exp. 2, mRNA expression levels of *Acta2*, *Tgfb1*, *Mmp9*, and *Timp1* were lower after the injection of UC-MSC-CM than in the DSS alone group; however, these differences were not statistically significant. This finding points to ECM remodeling with a bias toward anti-fibrogenic processes in the colon. In contrast to TGF-β1 and TIMP-1, which act as fibrogenic molecules in intestinal fibrosis, MMP-9 is generally thought to have an anti-fibrogenic effect due to its collagen proteolytic activity. However, it can also induce fibrogenesis by releasing or activating various pro-fibrotic molecules sequestered in the ECM by proteolytic degradation and mediating epithelial-mesenchymal transition, a process in which epithelial cells undergo phenotypic metamorphosis into myofibroblasts [75]. In addition, other studies have shown that MSCs reduce intestinal or renal fibrosis by decreasing MMP-9 expression or activity [42, 75, 76].

We investigated the anti-fibrotic effects of UC/PL-MSC-CM in a late-phase treatment model (Exp. 1), where UC/PL-MSC-CM was injected from Day 50, and an early-phase treatment model (Exp. 2), where UC/PL-MSC-CM was injected from Day 29. In Exp. 2, UC-MSC-CM showed more significant anti-fibrotic effects based on relative weight (%), colon weight/length, collagen quantification by a hydroxyproline assay, and Procol1A1-positive area. In Exp. 2, PL-MSC-CM also showed more effective anti-fibrotic effects based on measurements of the colon length and thickness of the submucosa/muscularis propria. Overall, UC/PL-MSC-CM treatment was more effective in curing fibrosis in the early phase of chronic DSS-induced intestinal fibrosis than in the late phase. These findings are consistent with those of previous studies revealing that anti-fibrotic intervention is more effective when initiated early in the course of the disease. Previously, early eradication of the fibrotic stimulus suppressed intestinal fibrosis, while late eradication did not prevent fibrotic progression [3]. Another study has shown that an anti-fibrotic molecule administered prophylactically is highly effective in slowing the progression of intestinal fibrosis, while administration during the late phase of fibrosis is less effective in clearing fibrosis [77]. Overall, our findings suggest that early UC/PL-MSC-CM treatment may be more effective than late treatment during intestinal fibrosis.

Co-culture with the UC/PL-MSC-CM significantly inhibited TGF-β1-mediated fibrogenesis in the HIMFs, as revealed by decreased ECM (Procol1A1, FN) and contractile protein (α-SMA) expression, along with a decrease in Smad2 phosphorylation. Several studies have shown that the UC/PL-MSC-CM inhibits fibroblast differentiation into myofibroblasts and reduces ECM and α-SMA expression in myofibroblasts [78, 79]. In addition, the discovery of reduced Smad2 phosphorylation in response to TGF-β1 treatment with the UC/PL-MSC-CM suggests that the anti-fibrogenic effects may occur via Smad-dependent mechanisms. UC-MSCs, but not PL-MSCs, decrease the TGF-β1-induced phosphorylation of Smad2 and Smad3 [23]. In addition, UC-MSC-derived exosomes suppress Smad2 activation and reduce TGF-β-induced α-SMA expression in skin fibroblasts [80].

In this study, we compared the anti-fibrotic effects of MSC-CM derived from two different sources, the UC and PL. Immunomodulatory effects are greater for UC-MSCs than for PL-MSCs. This is because UC-MSCs have higher expression of immunomodulatory factors, such as TGF-β, IL-10, and IDO (indoleamine 2,3-dioxygenase), all of which play important roles in immune regulation and inflammation suppression [81–84]. Additionally, a comparison of MSCs from various sources has shown that UC-MSCs exhibit the most significant immunosuppressive effects and the highest proliferative and differentiation potential [85]. Previously, we found that UC-MSCs exhibit pronounced anti-fibrotic activities in HIMFs [23]. Similarly, compared with the effects of PL-MSC-CM, UC-MSC-CM demonstrated more potent inhibitory effects in the chronic DSS-induced intestinal fibrosis model as well as on TGF-1-induced Procol1A1, FN, and α-SMA expression in HIMFs and the protein expression of MRTF-A, SRF, and RhoA. Thus, these findings suggest that UC-MSC-CM has more potent anti-fibrotic activity than that of PL-MSC-CM.

The Rho/ROCK/Actin/MRTF/SRF signaling axis, a Smad-independent pathway, is a key pathway involved in multiple types of solid organ fibrosis, including intestinal fibrosis [86–89]. RhoA, a GTPase from the Rho family, promotes G-actin integration into F-actin. Furthermore, RhoA-mediated ROCK activation inhibits F-actin depolymerization [90]. F-actin polymerization results in the release of G-actin-bound MRTF-A, which translocates to the nucleus and interacts with SRF, inducing the expression of fibrogenic genes, such as collagen 1A1 and α-SMA [9, 11]. Recent studies have shed light on the role of Rho/ROCK/Actin/MRTF/SRF signaling in intestinal fibrosis [9, 91, 92]. Our previous study has revealed that the anti-fibrogenic effects of UC/PL-MSCs are mediated via the Rho/MRTF/SRF signal pathway in HIMFs [23]. In this study, co-culture with UC/PL-MSC-CM suppressed ECM and α-SMA expression in HIMFs by attenuating TGF-β1-induced MRTF-A and SRF expression and inhibiting MRTF-A nuclear translocation. These findings indicate that the anti-fibrotic effects of UC/PL-MSC-CM may be mediated via a mechanism involving the MRTF/SRF pathway. We assessed RhoA, ROCK1, and ROCK2 expression in HIMFs to determine whether UC/PL-MSC-CM affects factors upstream of the MRTF/SRF pathway. UC/PL-MSC-CM reduced MRTF-A and SRF expression, while suppressing the expression of RhoA, ROCK1, and ROCK2. The Rho/ROCK signaling pathway is critical in MRTF/SRF transcriptional activation [11]. These findings imply that the UC/PL-MSC-CM exerts an anti-fibrotic effect by modulating the upstream TGF-β pathway, resulting in a broader impact beyond the selective inhibition of MRTF/SRF and interfering with the Rho/ROCK pathway.

## Conclusions

**Fig. 8:**
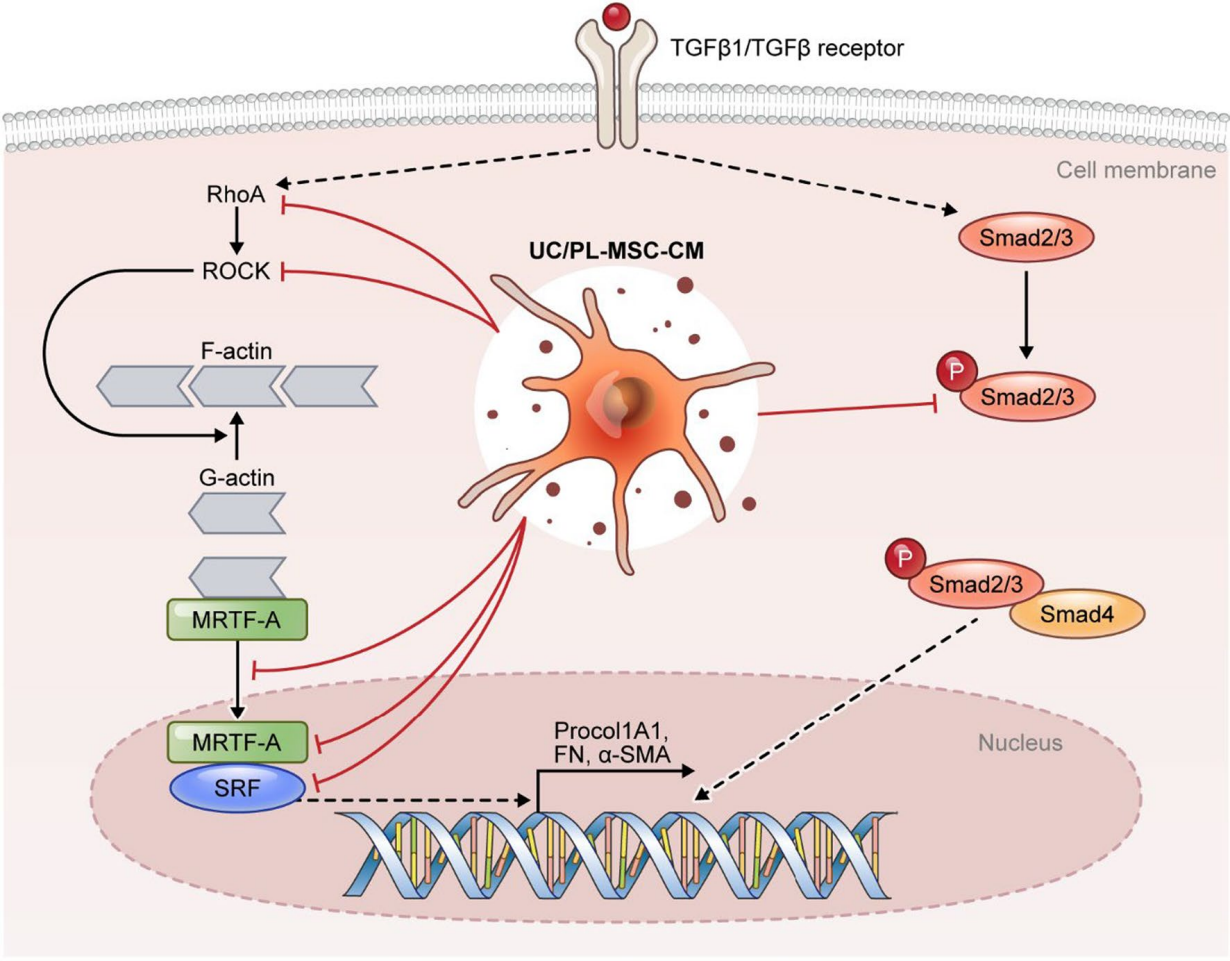
Model of the proposed mechanism of action of UC/PL-MSC-CM on TGF-β1-induced fibrogenic activation in HIMFs. TGF-β1 stimulates the expression of Procol1A1, FN, and α-SMA via the activation of the MRTF-A/SRF signaling and Smad2/3. UC/PL-MSC-CM counteract TGF-β1-induced fibrogenic responses by inhibiting the RhoA/MRTF-A/SRF signaling and Smad2/3 pathways

In this study, we observed that human UC/PL-MSC-CM exhibited anti-fibrotic properties in a murine model of intestinal fibrosis. The primary mechanisms underpinning these effects appear to be the suppression of collagen synthesis and the downregulation of α-SMA expression. Furthermore, UC/PL-MSC-CM showed a remarkable capability in mitigating TGF-β1-induced fibrogenic activation in HIMFs through the inhibition of the Rho/MRTF/SRF signaling pathway, as depicted in Fig. 8. Notably, the anti-fibrogenic effect was more pronounced in the UC-MSC-CM treatment or when applied in the early-phase of the model. Consequently, UC/PL-MSC-CM may emerge as a novel therapeutic approach for intestinal fibrosis. Nevertheless, further studies are essential to elucidate the precise mechanisms and identify the key anti-fibrotic constituents within MSC-CM, in order to confirm these findings and refine the therapeutic application of UC/PL-MSC-CM.

### Supplementary Information

**Additional file 1**. Supplementary figures.

**Additional file 2**. Supplementary methods and figure legends.

## Data Availability

All reported data have been obtained from experiments performed in the author’s laboratory. The dataset generated during the present study is available upon reasonable request from the corresponding authors (Prof. Jee Hyun Kim or Prof. Jun Hwan Yoo).

## Abbreviations

HIMFs: Human primary intestinal myofibroblasts
DSS: Dextran sulfate sodium
IBD: Inflammatory bowel disease
CD: Crohn’s disease
GI: Gastrointestinal
ECM: Extracellular matrix
α-SMA: α-Smooth muscle actin
TGF-β1: Transforming growth factor
G-actin: Promoting globular actin
F-actin: Filamentous actin
MRTF-A: Myocardin-related transcription factor A
SRF: Serum response factor
MSCs: Mesenchymal stem cells
UC/PL-MSCs: Umbilical cord and placenta-derived mesenchymal stem cells
CM: Conditioned medium
EVs: Extracellular vesicles
MMP: Matrix metalloproteinases
IL-10: Interleukin-10
HGF: Hepatocyte growth factor
miRNAs: MicroRNAs
α-MEM: α-modified minimal essential medium
FBS: Fetal bovine serum
FGF4: Fibroblast growth factor-4
FACS: Fluorescence-activated cell sorting
SEM: Standard error of the mean
FN: Fibronectin
hucMSC-Ex: Human UC MSC-derived exosomes
