# Supplementary material for: Human umbilical cord/placenta mesenchymal stem cell conditioned medium attenuates intestinal fibrosis in vivo and in vitro
Source: Stem Cell Res Ther. 2024 Mar 7;15:69. doi: 10.1186/s13287-024-03678-4 (PMC10921617; doi:10.1186/s13287-024-03678-4)
Supplement: Supplementary file 1 — Additional file 1. Supplementary figures. [file 13287_2024_3678_MOESM1_ESM.pptx]

## Slide 1
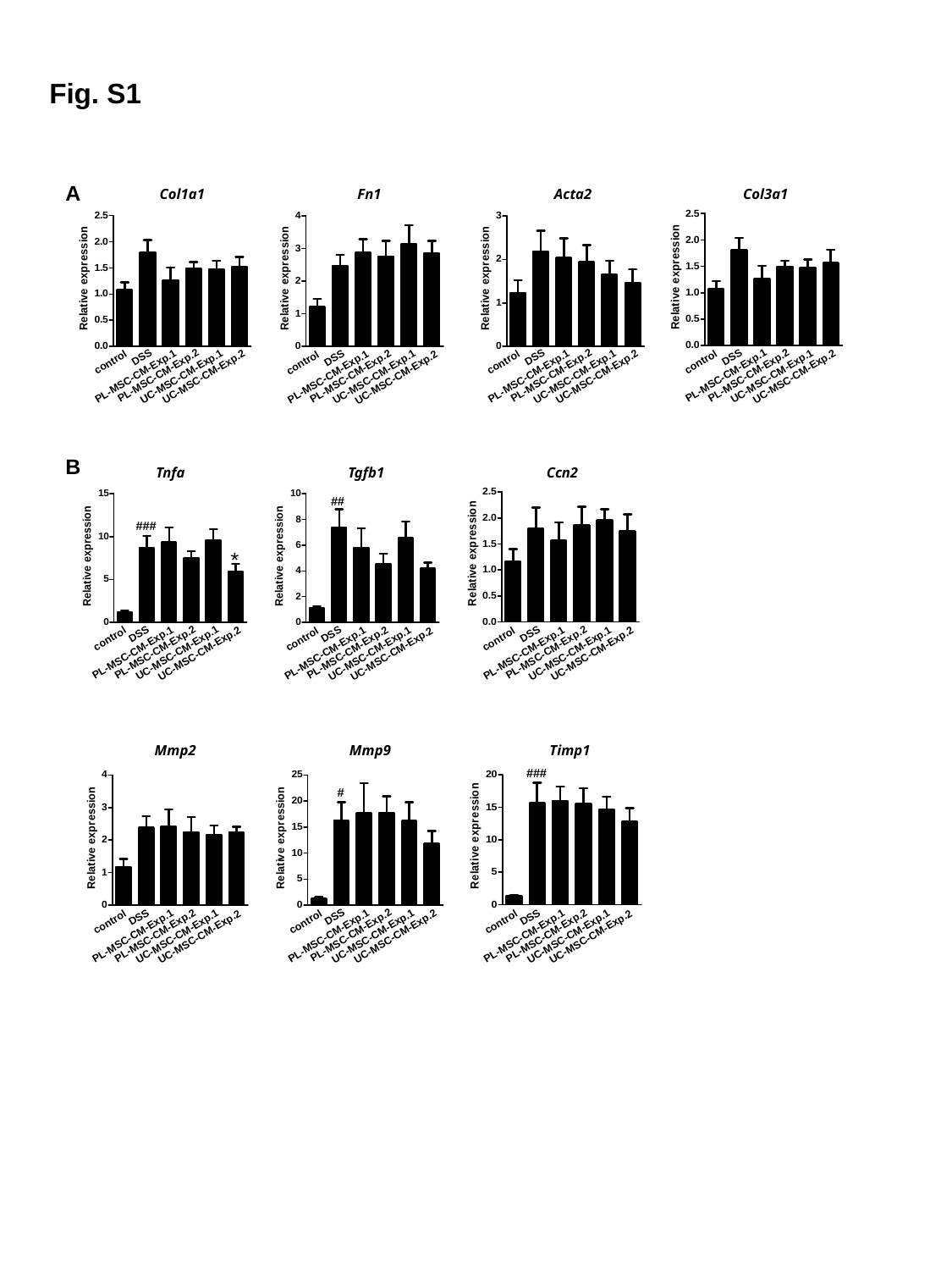

Fig. S1
A
Col1a1
Fn1
Acta2
Col3a1
DSS
control
PL-MSC-CM-Exp.2
PL-MSC-CM-Exp.1
UC-MSC-CM-Exp.1
UC-MSC-CM-Exp.2
DSS
control
PL-MSC-CM-Exp.2
PL-MSC-CM-Exp.1
UC-MSC-CM-Exp.1
UC-MSC-CM-Exp.2
DSS
control
PL-MSC-CM-Exp.2
PL-MSC-CM-Exp.1
UC-MSC-CM-Exp.1
UC-MSC-CM-Exp.2
DSS
control
PL-MSC-CM-Exp.2
PL-MSC-CM-Exp.1
UC-MSC-CM-Exp.1
UC-MSC-CM-Exp.2
B
Tnfa
Tgfb1
Ccn2
##
###
*
DSS
control
PL-MSC-CM-Exp.2
PL-MSC-CM-Exp.1
UC-MSC-CM-Exp.1
UC-MSC-CM-Exp.2
DSS
control
PL-MSC-CM-Exp.2
PL-MSC-CM-Exp.1
UC-MSC-CM-Exp.1
UC-MSC-CM-Exp.2
DSS
control
PL-MSC-CM-Exp.2
PL-MSC-CM-Exp.1
UC-MSC-CM-Exp.1
UC-MSC-CM-Exp.2
Mmp2
Mmp9
Timp1
###
#
DSS
control
PL-MSC-CM-Exp.2
PL-MSC-CM-Exp.1
UC-MSC-CM-Exp.1
UC-MSC-CM-Exp.2
DSS
control
PL-MSC-CM-Exp.2
PL-MSC-CM-Exp.1
UC-MSC-CM-Exp.1
UC-MSC-CM-Exp.2
DSS
control
PL-MSC-CM-Exp.2
PL-MSC-CM-Exp.1
UC-MSC-CM-Exp.1
UC-MSC-CM-Exp.2

## Slide 2
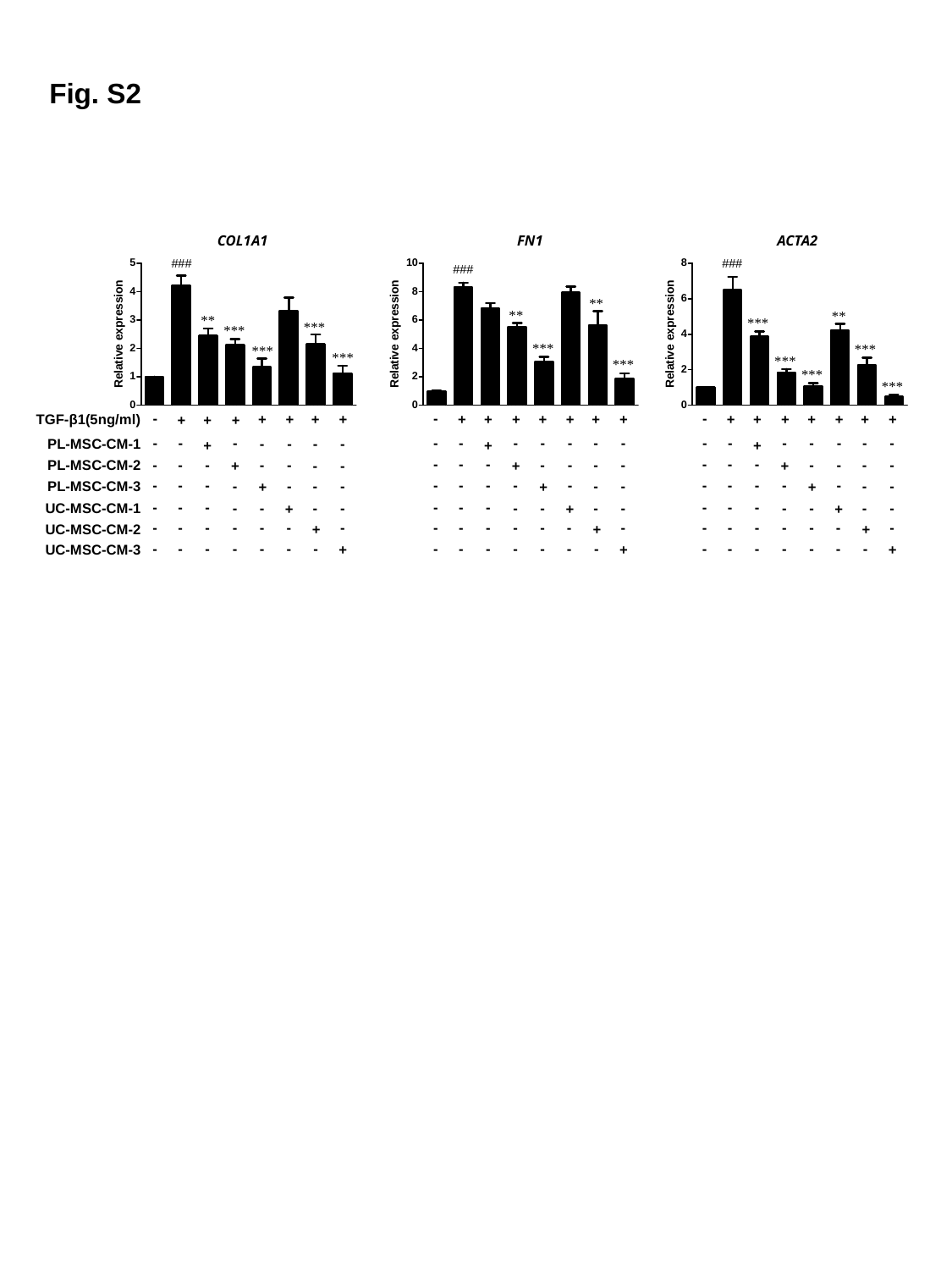

Fig. S2
###
###
###
**
**
**
**
***
***
***
***
***
***
***
***
***
***
***
-
-
-
+
+
+
+
+
+
+
+
TGF-β1(5ng/ml)
+
+
+
+
+
+
+
+
+
+
+
+
+
-
-
-
-
-
-
-
-
-
-
-
-
-
-
-
-
-
-
-
-
-
PL-MSC-CM-1
+
+
+
-
-
-
-
-
-
PL-MSC-CM-2
-
-
-
-
-
-
-
-
-
-
-
-
-
-
-
+
+
+
-
-
-
-
-
-
-
-
-
-
-
-
-
PL-MSC-CM-3
-
-
-
-
-
-
-
-
+
+
+
-
-
-
-
-
-
-
-
-
UC-MSC-CM-1
-
-
-
-
-
-
-
-
-
-
-
-
+
+
+
-
-
-
-
-
-
-
-
-
-
-
-
-
-
-
-
-
-
-
-
-
UC-MSC-CM-2
+
+
+
-
-
-
-
-
-
-
-
-
-
-
-
-
-
-
-
-
-
-
-
-
+
+
+
UC-MSC-CM-3
COL1A1
FN1
ACTA2

## Slide 3
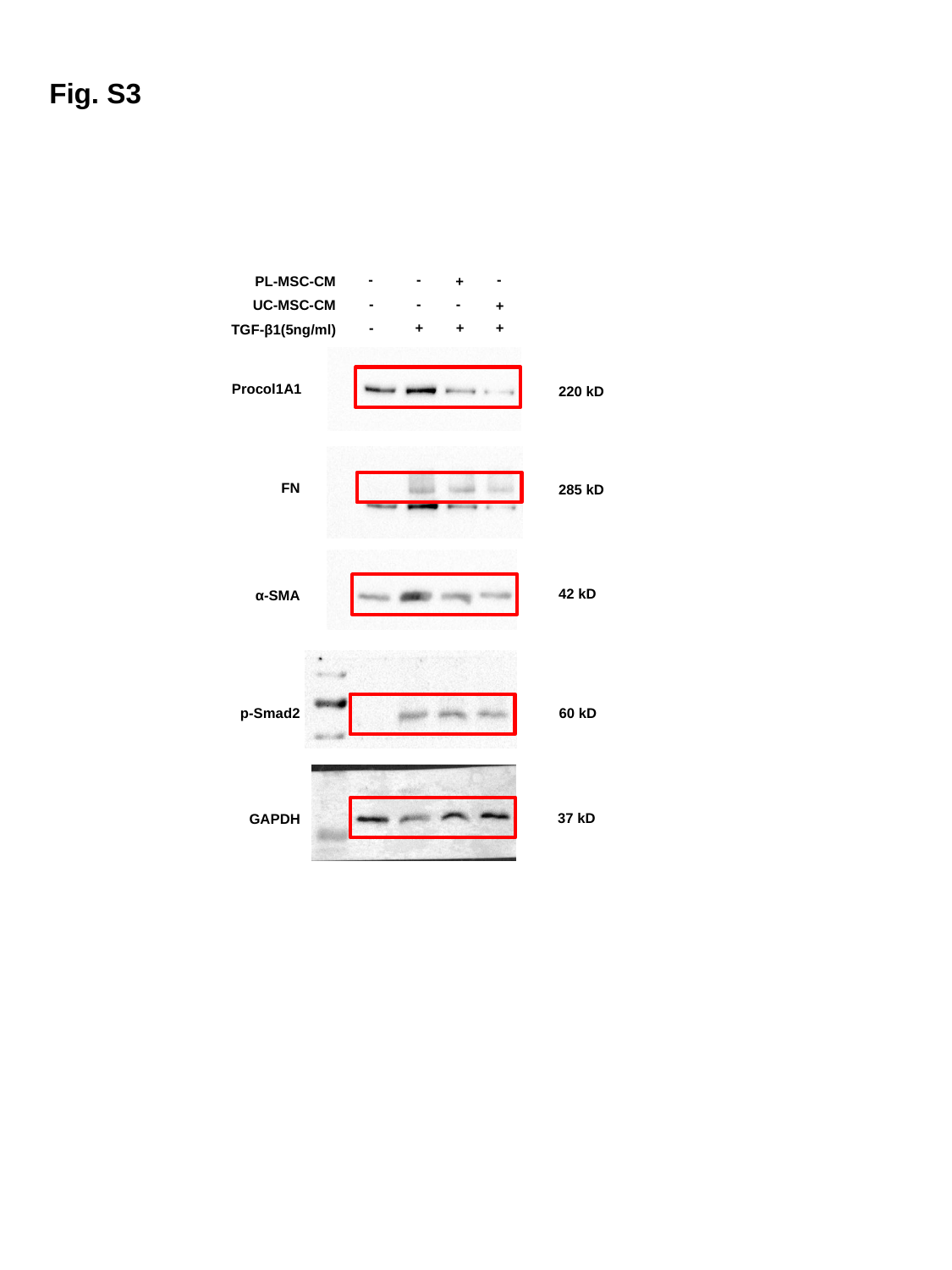

Fig. S3
-
-
-
PL-MSC-CM
+
-
-
-
UC-MSC-CM
+
-
+
+
+
TGF-β1(5ng/ml)
Procol1A1
220 kD
FN
285 kD
42 kD
α-SMA
p-Smad2
60 kD
37 kD
GAPDH

## Slide 4
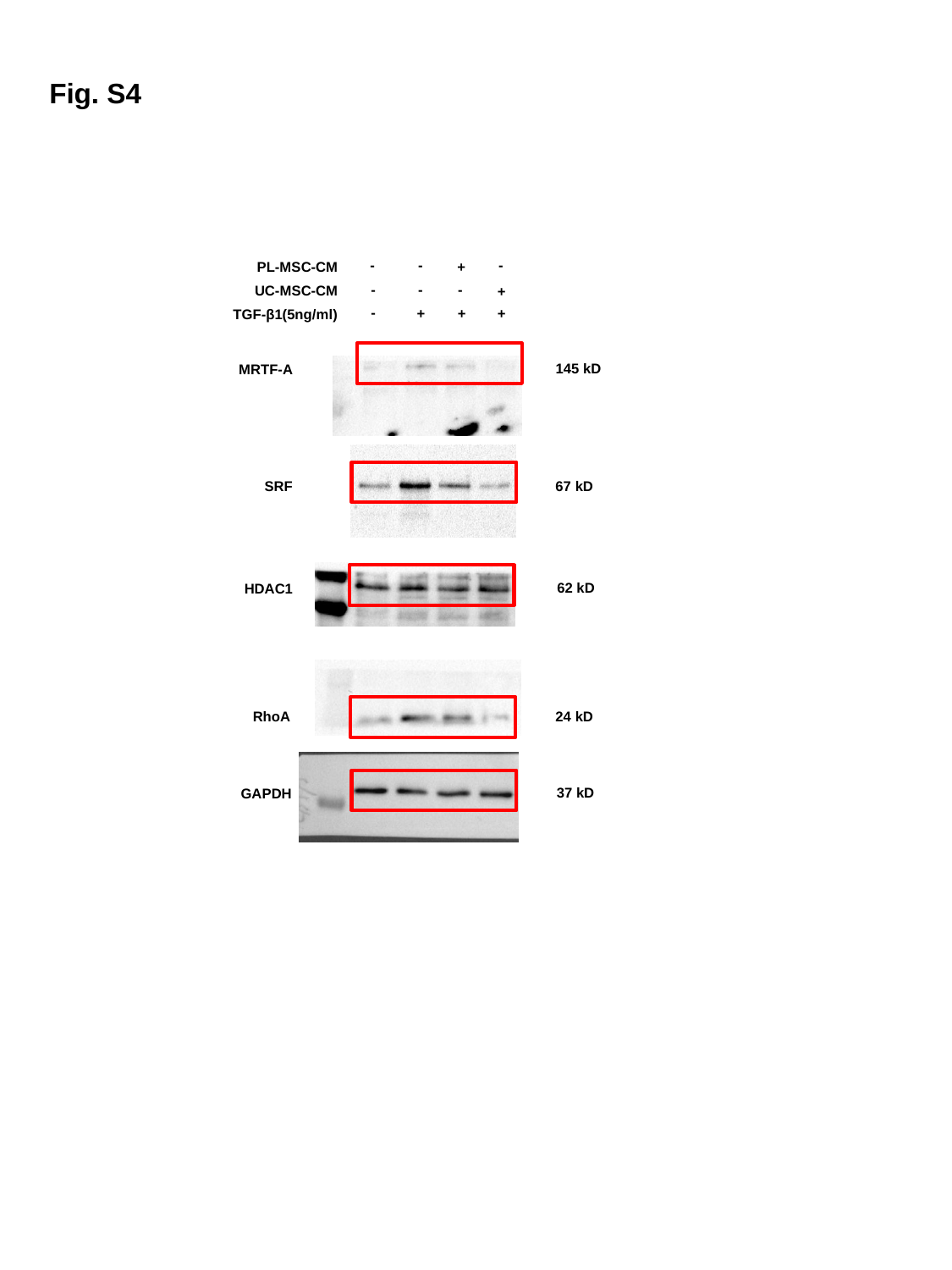

Fig. S4
-
-
-
PL-MSC-CM
+
-
-
-
UC-MSC-CM
+
-
+
+
+
TGF-β1(5ng/ml)
145 kD
MRTF-A
67 kD
SRF
62 kD
HDAC1
RhoA
24 kD
37 kD
GAPDH
