# Supplementary material for: Human umbilical cord/placenta mesenchymal stem cell conditioned medium attenuates intestinal fibrosis in vivo and in vitro
Source: Stem Cell Res Ther. 2024 Mar 7;15:69. doi: 10.1186/s13287-024-03678-4 (PMC10921617; doi:10.1186/s13287-024-03678-4)
Supplement: Supplementary file 2 — Additional file 2. Supplementary methods and figure legends. [file 13287_2024_3678_MOESM2_ESM.docx]

**Supplementary Methods**

**RNA isolation and real-time quantitative PCR (RT-qPCR) from colon tissue**

Total RNA was extracted from colon tissues using Trizol (Invitrogen) according to the standard protocol of the manufacturer and then purified via lithium chloride precipitation [1]. cDNA synthesis and RT-qPCR were performed as described in the "Material and methods" section. The specific primers for the collagen1A1 (Mm00801666_g1), fibronectin (Mm01256744_m1), α-SMA (ACTA2, Mm00725412_s1), collagen3a1 (Mm00802300_m1), TNF-α (Mm00443258_M1), TGF-β1 (Mm01178820_m1), CTGF (Mm01192933_g1), MMP2 (Mm00439498_m1), MMP9 (Mm00442991_m1), and TIMP (Mm01341361_m1) genes were purchased from Applied Biosystems (Foster City, CA).

**Reference**

1. Viennois E, Tahsin A, Merlin D. Purification of total RNA from DSS-treated murine tissue via lithium chloride precipitation. Bio-protocol. 2018;8(9):e2829-e.

**Supplementary Figures**

**Fig. S1 Colonic transcription levels of fibrosis-related genes.** **A** RT-qPCR analysis of the relative mRNA expression of collagen1A1 (*Col1a1*), fibronectin (*Fn1*), α-smooth muscle actin (*Acta2*), and collagen3A1 (*Col3a1*). The data were normalized to GAPDH expression and expressed as relative values compared to the control (n = 3). **B** RT-qPCR analysis of the relative mRNA expression of TNF-α, TGF-β1, CTGF, MMP2, MMP9, and TIMP (*Tnfa, Tgfb1, Ccn2, Mmp2, Mmp9,* and *Timp1*). The data were normalized to GAPDH expression and expressed as relative values compared to the control (n = 3). Data are expressed as the means ± SEM. # P < .05, ## P < .01 and ### P < .001 compared with control (ANOVA w/ Tukey).

**Fig. S2 UC/PL-MSC-CM inhibits TGF-β1-induced fibrogenic activation of HIMFs in a dose-dependent manner.** HIMFs were treated with TGF-β1 (5 ng/mL) and co-cultured with or without UC/PL-MSC-CM-1, -2, and -3, harvested for 1, 3, and 5 d, respectively. RT-qPCR analysis of the relative mRNA expression of collagen1A1 (*COL1A1*), fibronectin (*FN1*), and α-smooth muscle actin (*ACTA2*). The data were normalized to GAPDH expression and expressed as relative values compared to the control (n = 3).

**Fig. S3 Full-length blots for Fig. 4B.** Red boxes indicate the cropped blots shown in Fig. 4B. Membranes were often cut to enable blotting for multiple antibodies.

**Fig. S4 Full-length blots for Fig. 6B.** Red boxes indicate the cropped blots shown in Fig. 6B. Membranes were often cut to enable blotting for multiple antibodies.
